# Proof of Concept of Impact Detection in Composites Using Fiber Bragg Grating Arrays

**DOI:** 10.3390/s130911998

**Published:** 2013-09-09

**Authors:** Javier Gomez, Iagoba Jorge, Gaizka Durana, Jon Arrue, Joseba Zubia, Gerardo Aranguren, Ander Montero, Ion López

**Affiliations:** 1 Department of Communications Engineering, Faculty of Engineering, University of the Basque Country, Alda Urquijo, s/n. Bilbao 48013, Spain; E-Mails: gaizka.durana@ehu.es (G.D.); jon.arrue@ehu.es (J.A.); joseba.zubia@ehu.es (J.Z.); gerardo.aranguren@ehu.es (G.A.); 2 Aeronautical Technologies Center, Technologic Park of Alava, C/Juan de la Cierva 1, Miñano 01510, Alava, Spain; E-Mails: iagoba.jorge@ctaero.com (I.J.); ander.montero@ctaero.com (A.M.); ion.lopez@ctaero.com (I.L.)

**Keywords:** structural health monitoring, fiber Bragg grating, remote sensing, optical sensors

## Abstract

Impact detection in aeronautical structures allows predicting their future reliability and performance. An impact can produce microscopic fissures that could evolve into fractures or even the total collapse of the structure, so it is important to know the location and severity of each impact. For this purpose, optical fibers with Bragg gratings are used to analyze each impact and the vibrations generated by them. In this paper it is proven that optical fibers with Bragg gratings can be used to detect impacts, and also that a high-frequency interrogator is necessary to collect valuable information about the impacts. The use of two interrogators constitutes the main novelty of this paper.

## 1. Introduction

Nowadays, the use of sensors based on optical fibers [1] has increased considerably due to the inherent advantages of optical fibers, such as their low weight, their electromagnetic compatibility and the fact that, apart from their conventional applications, they can also be employed for unconventional purposes, e.g., as biosensors [2]. Traditionally, structural-health monitoring has been carried out by means of electromechanical or electroacustic systems [3], but fiber-based sensors and, especially, fiber Bragg gratings (FBGs) in optical fibers have gradually increased the technological applications of fiber devices [4] and they warrant consideration as sensors. One of the markets in which they are increasingly being used is the aerospace industry [5], due to their low weight, absence of distortion in communications systems and the fact that they cannot produce sparks that could cause fires, since the transmission medium is optical instead of electrical.

It has been reported in the literature that FBGs can detect variations in strain [6] and temperature [7] on a structure. Moreover, they are widely used in applications like structural-health monitoring. For this purpose, the tests are typically performed in a quasi-static way, by measuring relatively low displacement frequencies. The aim of this paper is to study the use of these Bragg gratings to detect changes in strain by measuring, at high frequency, the vibrations caused by low-speed impacts on an airplane structure [8]. Other objectives are to detect the position and the energy of the impact.

After carrying out the impact tests it is proven that optical fibers with Bragg gratings can reliably detect the impact of a piece of composite material on the surface. The position and the energy of impacts cannot be determined since it is necessary to use a high-frequency interrogator in order to collect all the information of the mechanical waves generated by the impact. Throughout this work two interrogators are used and it is verified, by means of the frequency analysis of the signals obtained, that the higher-frequency interrogator is the one that can provide more information about each one of the impacts. This constitutes the main contribution of this paper.

## 2. Experimental Section

To determine if the use of FBGs is effective in the detection of impacts, two arrays, each containing five Bragg gratings, are used. The reflection wavelengths (Bragg wavelengths) of the FBGs in each array are 1,526, 1,536, 1,546, 1,556 and 1,566 nm, with a sensitivity of 1.25 pm/µε and 10 pm/°C. The strain limit is ±5,000 με.

**Figure 1.**
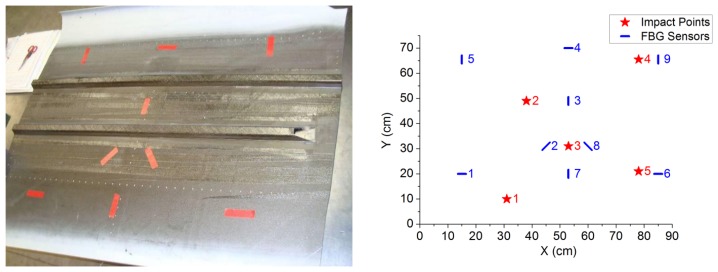
Marking and pre-allocation of the Fiber Bragg Grating on the bottom surface of the aircraft skin.

The specimen used to assess the effect of impacts on its surface is a piece of Carbon Fiber Reinforced Polymer (CFRP) with two stringers on the underside. The top side of the piece is finished with a white protecting coat (painted). The bottom, on which the FBGs were installed, has no special finishing, so this surface was more suitable to achieve a greater adhesion of the fibers (Figure 1). The size of the piece is 111 × 80–95 × 0.15 cm (length × width × thickness). The stringers were arranged in a parallel pattern. The piece is a part of the outer skin of a standard mass-produced aircraft.

Before mounting the FBGs, a preliminary study was conducted to determine the most effective means of positioning them, with the aim of measuring the variations of strain along as many directions as possible. Bragg gratings yield a good measurement response to changes in strain in the longitudinal direction of the optical fiber, but the response is only residual in the direction perpendicular to the optical fiber [9]. Among all the possibilities studied, the arrangement of FBGs that offered the largest amount of different usable orientations was chosen. This coincides with the arrangement of [10]. It consists in FBGs placed horizontally, vertically and diagonally (see Figure 1), but taking into account that two consecutive sensors should never lie in parallel orientations. Very near to the centre of the specimen, FBGs are arranged in the form of a star, as shown in Figure 1 both in the photograph of the left and in the diagram of the right. The advantage of placing the Bragg gratings in this way is that it allows us to detect the waves coming from an impact regardless of the location in which they happen.

**Figure 2.**
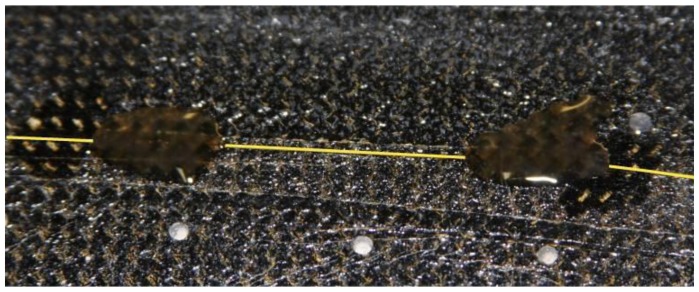
Detail of an installed Fiber Bragg Grating using epoxy as bond material.

A strict procedure was followed to mount the fiber on the specimen. First, the surface was cleaned with a degreaser to remove any traces of dirt or grease. Then, this surface was polished using 400 grit sandpaper and wiped again with a conditioner. Finally, the pH was adjusted to be optimal by means of a neutralizer. The bonding method followed is similar to that usually applied to strain gauges, and the result is shown in Figure 2, which a photograph of the mounted Bragg grating. The adhesive chosen was the M-Bond AE10, which is recommended by the manufacturer of FBGs for such applications.

Only nine of the 10 available FBGs in the two arrays of five Bragg gratings were used as structural sensors. The remaining one was employed to measure the temperature, with the purpose of compensating the effect of temperature fluctuations on the collected strain values. For that purpose, a tube made of polytetrafluroethylene (PTFE) of 1 mm of diameter was used to isolate that Bragg grating from the vibrations. By inserting that FBG in the tube, the fluctuations in the results yielded by it were only due to the temperature variations during the test, since they were not affected by vibrations, as was confirmed in the preliminary tests. Equation (1) was used to convert the data provided by the Bragg interrogator to longitudinal strain:

(1) $$\Delta ɛ=\frac{\Delta \lambda_{B}-\Delta \text{T}\cdot {\text{k}}_{T}}{k_{ɛ}}$$

where ∆*ε* is the difference in longitudinal strain, ∆*λ_B_* is the change in the Bragg wavelength corresponding to that Bragg grating, ∆T is the temperature variation, which was obtained from the isolated FBG, *k_T_* is the quotient between the shift in the Bragg wavelength and the change in temperature (pm/°C), and *k_ε_* is the strain constant (pm/µε).

The test procedure included five impact points, with different applied impact energies. It consisted in several series of three repetitions of the impact in the aforementioned points, varying the energy from one series to the next one. In Figure 1 we can see the positions of the Bragg gratings and the impact points considered. The sensors (S1–S9) are marked in black and the impact points are marked in red.

In Table 1 it is possible to check the different positions of the Bragg sensors and the impact points. The second column represents the x-coordinate (*i.e.*, in the horizontal axis), measured in cm, of the location of the sensor. The third column stands for the y-coordinate (*i.e.*, in the vertical axis) of that location. The last column is the angle made by the direction of the sensor with the x-axis. The orientation of the FBG in a certain direction does not have any effect on the measurement of the strain.

**Figure 3.**
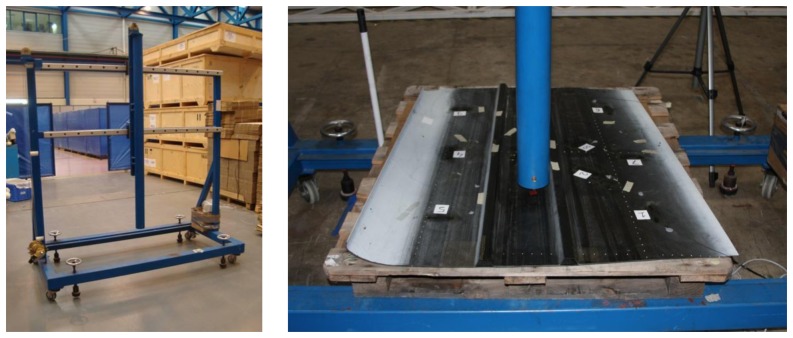
Certified impact machine to be used with aeronautical specimens.

For the impact generation, an impact machine was used. The device consisted of a vertical tube inside which a solid mass fell towards the specimen. The tube was mounted vertically on a mobile frame. The impact energy can be calculated simply from the mass and the height difference, disregarding the friction force. It is just the product of the mass, the acceleration of gravity and the height. The employed mass could be interchanged to increase or decrease the impact energy in the test. Every mass had a protrusion on the bottom consisting in a semi spherical shape with a radius of about 1.5 cm. The machine has aeronautical certification and is commonly used by the Aeronautical Technologies Centre for impact testing. In Figure 3 we can see the impact machine before placing the specimen. To hold the specimen in the machine, we used a pallet made of wood.

The rest of the assembly consisted of a SM130-200 Bragg interrogator. The basic features of this model are a wide range of inspection wavelengths, from 1,510 to 1,590 nm, a data acquisition rate of 100 Hz, two channels with up to 80 sensors per channel and a resolution of 2 pm. The interrogator collects and sends data via Ethernet to a Labview-based program installed on a laptop.

## 3. Results and Discussion

A low-speed impact produces surface mechanical waves that travel along the whole structure, generating strain fluctuations that can be measured by FBGs. In our tests, impact location was accomplished by triangulation, using the arrival time differences, at each one of the sensors, for the primary wave produced by the impact. The test conditions are summarized in Table 2. The test energy of each series of impacts was successively increased from 0.245 J to 29.4 J, by changing the employed masses and the heights of fall. The impact energy can be calculated by multiplying the mass, the acceleration of gravity and the height. The mass employed in each test is specified in the second column of Table 2, and the height is in the third column. The last column represents the impact energy.

As explained in Section 2, we carried out three repetitions for each measurement of the five predefined points of impact. Visual inspections and thermograph analyses were done after each series of impacts, to check that the specimen had not experienced a significant deterioration. Until the test number 8 the specimen was only supported on the pallet. From that test onwards, it was tied with two clamps to the pallet to avoid its movement while being impacted. The tests were stopped after noticing that, during the test number 13, the sample had begun to show signs of delamination.

**Figure 4.**
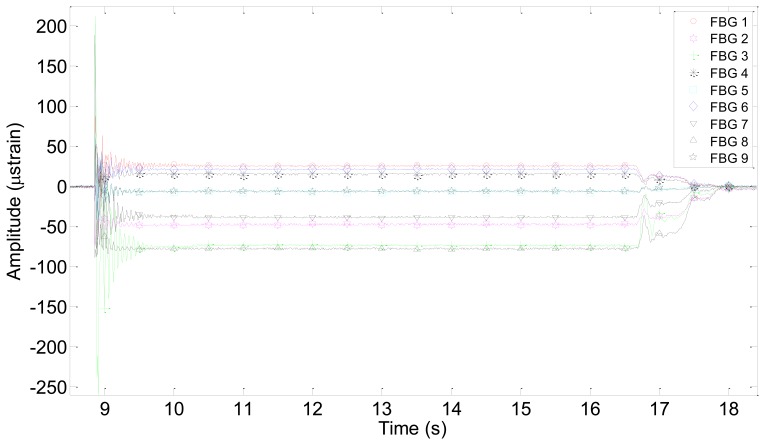
Results obtained with an impact of 4.9 J (Test number 8) at impact point 3.

As an example, Figure 4 shows the results obtained for impact point No. 3 during test No. 8. Most of the graphs have very similar shapes, although, as expected, the amplitude increases when increasing the impact energy. In all cases, the response of the FBGs is a damped oscillation (a sinusoidal-like curve with an exponential decrease of its amplitude with time).

In Figure 4, it can be noticed that all the graphs tend to a constant value different from zero when the impact is almost completely dumped until the mass is lifted again. This effect can be explained from the mechanical and manual operation of the impact device. After dropping the impact mass and before picking it to its initial position, the mass is permanently pressing the sample, because there is not an automatic device that lifts the mass to the initial position and manual action is required. Therefore, a deformation is generated in the specimen in the time interval in which the mass is on it. The deformation during that time interval was employed to analyze the static deformations of the specimen as a function of the value of the mass and of its location on the sample.

Tables 3 and 4 summarize the relationship between the residual strain when the mass is on the impact point, the distance from that point to the Bragg sensor and the angle between the orientation of the Bragg sensor and the straight line to the impact point. Both tables correspond to results obtained using Impact Point 1.

**Figure 5.**
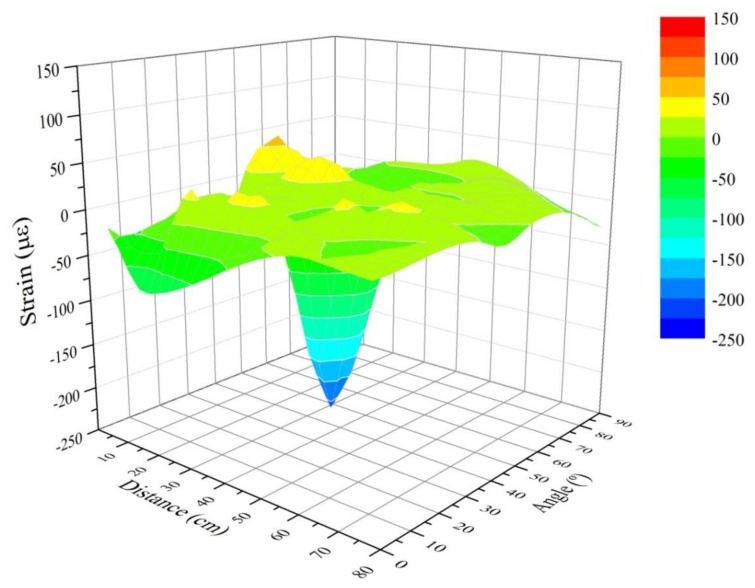
Residual strain with the mass of 2.5 kg.

**Figure 6.**
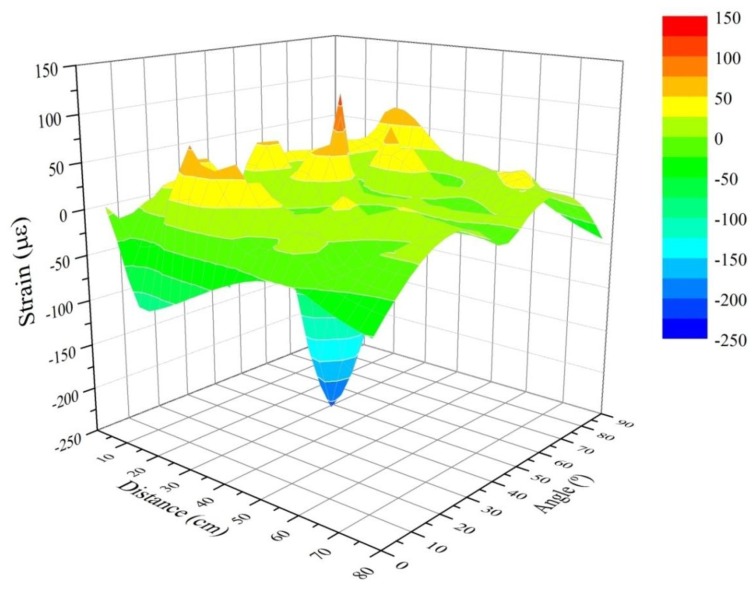
Residual strain with the mass of 10 Kg.

Figure 5 shows, graphically, the results corresponding to the residual strain before an impact test for all the impact points using the mass of 2.5 kg. Figure 6 contains the results for the 10 kg mass. We expected to get the highest values near to the Bragg sensor (0 cm) and longitudinally (0° angle). However due to the inherent physical properties of the CFRP sample, which make the structure behave as an anisotropic body, the strain in different places does not correspond to a theoretical distribution in a simpler isotropic material (such as a metallic sample). The fact that the overall structure of the sample (not the composition of the layers) includes stringers and reinforced areas also enhances this non-linearity of the responses received by the sensors when measuring the arrival time of the wave created by the impacts. Therefore, we can obtain wave-arrival-time measurements that are different from the expected ones in an isotropic material. This leads us to believe that the results obtained and reflected in the next charts (Figures 5 and 6) are correct. The next Figure 6 shows the results corresponding to all impact points and the mass of 10 kg.

**Figure 7.**
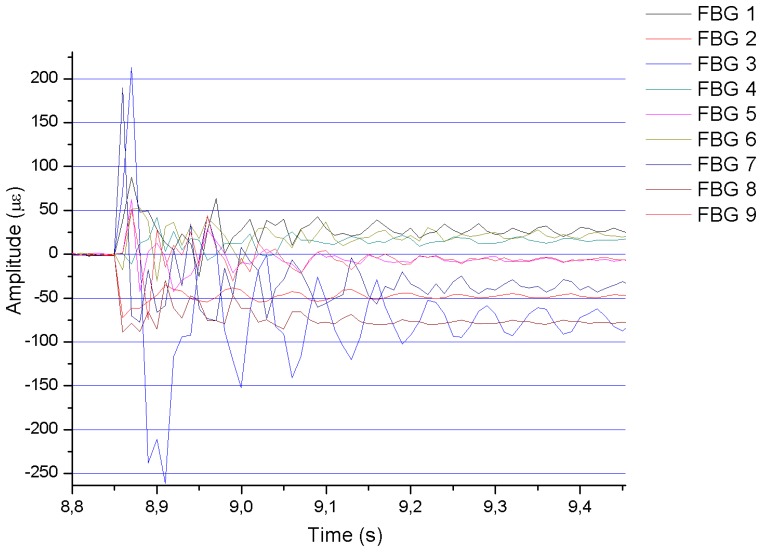
Detail of a chart containing the data resulting from an impact of 4.9 J (Test 8) at impact point 3.

Figure 7 shows the results corresponding to an impact in detail. Notice that, in this case, it is impossible to distinguish the exact moment in which the strain waves reach each of the FBGs, which makes it very difficult to determine the distance from each FBG to the impact point with accuracy. The problem arises because the speed of our interrogator to detect variations in the wavelength at each Bragg grating is too low to clearly detect the waves generated by the impact. In Section 3.1 we discuss in detail this problem and we give a solution to this question.

**Figure 8.**
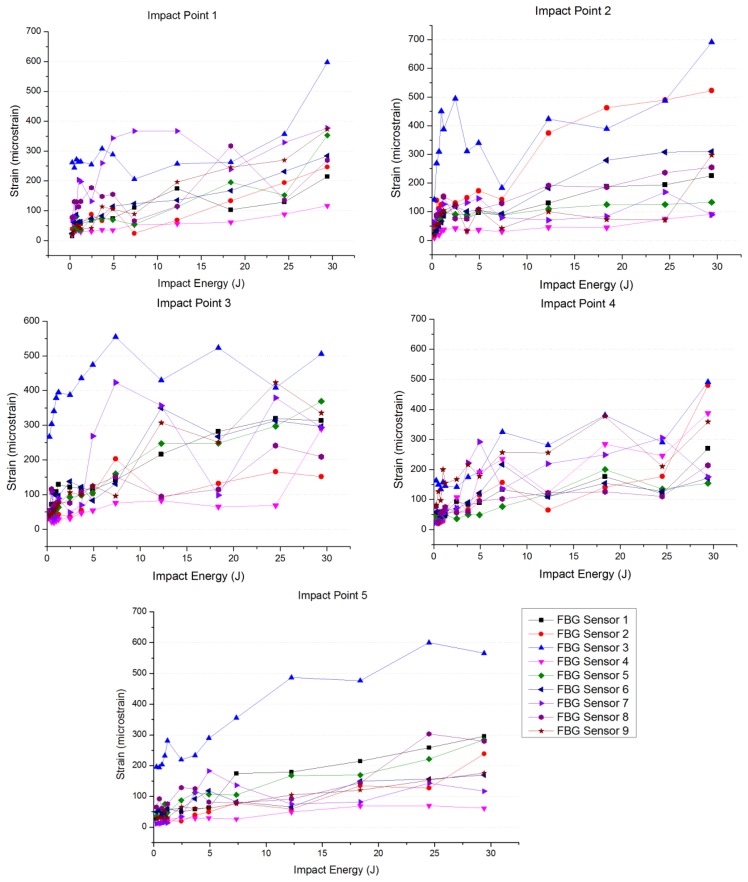
Relation between the impact energy and the strain measured.

On the other hand, the amplitude corresponding to the strain signal detected by each Bragg grating has been related to the energy of the performed impacts. As a representation of this relationship, we have obtained the graph shown in Figure 8. This graph represents the difference between the maximum and minimum strain values at each FBG sensor, the impact point being the same in all curves, when using increasing values of the energy applied in the impacts. It can be observed that the amplitude of the measured strain increases as the impact energy does. In all the performed tests, the maximum amplitude is collected at Bragg grating 3, located just in the center area of the specimen.

### 3.1. Optimization of Detection of Impacts Using a High Frequency Interrogator

To triangulate the position of the impact and its energy, it is necessary to fully recognize the exact moment when the primary wave generated by the impact reaches each Bragg grating and, through simple processing, to calculate the position. In order to know the applied energy, it is necessary to obtain exactly the maximum strain of each Bragg grating after the impact and, knowing the position of the impact, we must correct the calculation taking into account the attenuation of the mechanical wave from the impact point to the position of the Bragg grating and the relationship between the propagation direction and the orientation of the Bragg grating.

During the tests, it was very difficult to distinguish the point from which the impact waves were arriving to each Bragg grating. Besides, it was difficult to observe the moment when the signal of the impact arrived at each one of the FBGs. That meant that the problem was the sampling rate of our equipment. In order to confirm that this was the problem, instead of any other reason, another interrogator with a higher data acquisition rate was applied [10]. This interrogator worked at up to 20 kHz of data-acquisition rate. The only problem found during its use was the following: with the 100 Hz interrogator all the Bragg gratings were visible and gave data, but with the 20 kHz interrogator only two of our gratings were visible.

With the data obtained using the 20 kHz interrogator and knowing the impact points and the position of the Bragg gratings it is possible to obtain the propagation speed of the mechanical waves in the specimen.

**Figure 9.**
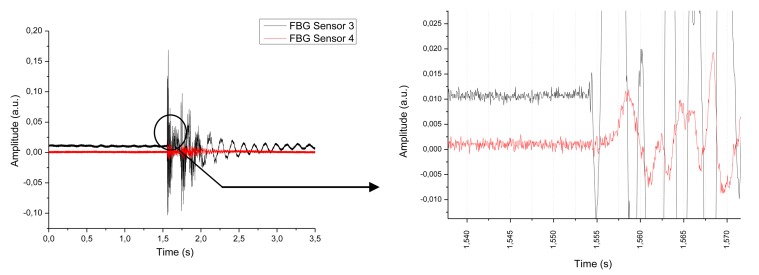
Detail of the wavefronts received by each sensor (Impact point 3, Fiber Bragg Gratings 3 and 4).

From these data, we can know the time between the arrivals of the wavefronts generated by the impact to each Bragg grating sensor. In Figure 9 we show the results of an impact for a fall of 10 cm to impact point 3 and a mass of 2.5 kg.

The time slot for the arrival of the wavefront to each sensor is 1.45 ms, having the reference of 10% of signal variation, and the travelled distance is 21 cm. The corresponding speed of the mechanical wave is 144.82 m/s. Although the typical speed of a mechanical wave in a CFRP is about 3,000 m/s, this speed could change according to many factors like humidity, composite layer arrangement and so on [11].

**Figure 10.**
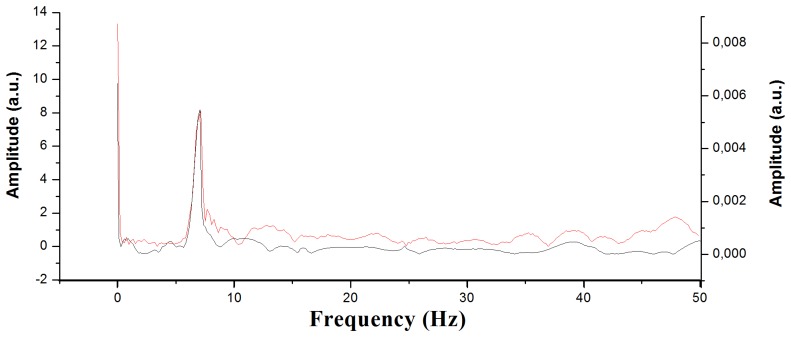
Fast-Fourier-Transform graph with the 100 Hz interrogator (red) and the 20 kHz interrogator (black).

To demonstrate the improvement obtained when using an interrogator with a higher sampling speed, the Fast Fourier Transform (FFT) was applied to both signals. The goal was to determine which part of the impact signal was not collected when using a low-frequency interrogator. The FFT limits for the data collected by the interrogator of 100 Hz were from 0 to 50 Hz, and the limits extended up to 10 kHz for the 20 kHz interrogator. The FFT serves us to know if there are non-negligible spectral components of the signal between 50 Hz and 10 kHz. If not, a 100-Hz interrogator would yield the same results as a 20-kHz one. However, the FFT results clearly show that the high-frequency values cannot be neglected, which means that information is lost with the low-frequency interrogator. When comparing the results obtained by the two interrogators applying the FFT up to 50 Hz, we can create graphs such as Figure 10. The data obtained are basically identical; the only difference lies in the range, due to the fact that the measuring method is different in both interrogators.

**Figure 11.**
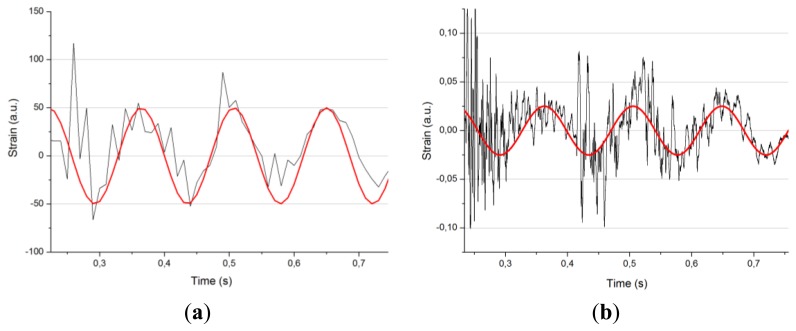
Strain value graph recorded at 100 Hz by means of the MicronOptics interrogator (**a**) and data for a same location and energy impact obtained with a 20 KHz interrogator (**b**). In red there is a sinusoidal pattern of 7 Hz to serve as a help when identifying the fundamental frequency of the studied signal.

According to the FFT results, there is a 7 Hz tone in the signal. This may be related to some fundamental vibration frequency of the specimen. Apart from this tone, which can be easily observed, we have numerous variations in the signal at higher frequencies. In Figure 11 we can see the differences between the data collected by the 100 Hz interrogator (left) and the data collected at 20 kHz (right).

**Figure 12.**
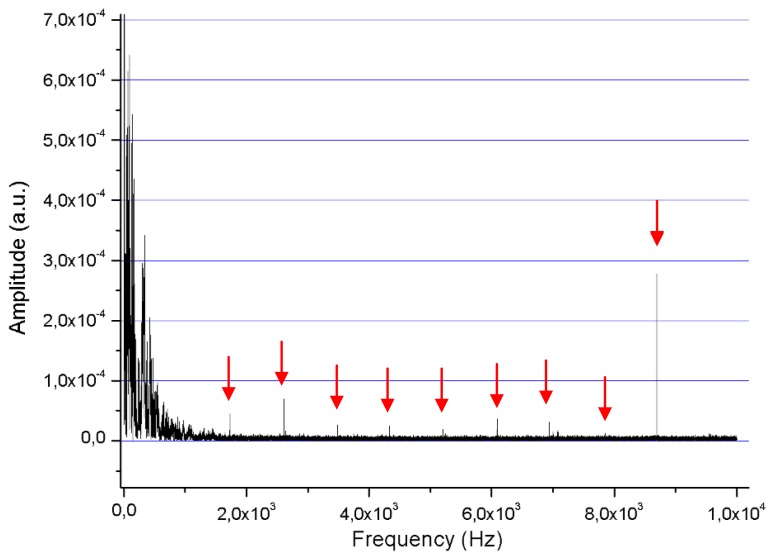
Fast-Fourier-Transform graph for the data collected by the 20 kHz interrogator. Red arrows indicate the most interesting values collected.

In addition to the FFT data provided above, the 20 kHz interrogator allows performing a 10 kHz FFT where values above 50 Hz appear (Figure 12). This is the highest limit that is possible to reach with the first interrogator applied. The frequency is especially important when considering the total effect of impact.

The peak frequencies, marked in red in the previous figure, are the following: 1,726 Hz, 2,606 Hz, 3,485 Hz, 4,334 Hz, 5,211 Hz, 6,091 Hz, 6,937 Hz, 7,851 Hz and 8,697 Hz. These frequencies correspond to the natural frequencies in torsional and flexural modes [12], although the calculus of the frequencies of this complicated structure is out of the scope of the present paper.

## 4. Conclusions

We have shown that fiber Bragg gratings written in optical fibers are suitable to detect an impact on the surface of an aircraft structure. In comparison with the traditional detection methods employed so far, this method presents advantages, such as the low weight of optical fibers, their electromagnetic compatibility and their protection against possible sparks. Moreover, the applications of fibers with Bragg gratings are numerous, since they can also be employed for other types of structures, for example for nuclear power plants or for underwater pipes. When it is necessary to detect the exact position of an impact and the created energy, a high-speed Bragg interrogation system should be used for the data acquisition. In this paper, we have used a 100 Hz interrogation system and a high-performance one of up to 20 kHz, and the results indicate that, as a rule of thumb, the interrogation system should be of several hundreds of kHz, at least, to detect and completely characterize the impact.

## Figures and Tables

**Table 1.** Fiber-Bragg-Grating Sensor and Impact Locations.

	**Position X (cm)**	**Position Y (cm)**	**Angle (related to the x-axis)**
Sensor No. 1	15	20	0°
Sensor No. 2	45	31	45°
Sensor No. 3	53	49	90°
Sensor No. 4	53	70	0°
Sensor No. 5	15	65.5	90°
Sensor No. 6	85	20	0°
Sensor No. 7	53	20	90°
Sensor No. 8	60	31	135°
Sensor No. 9	85	65.5	90°
Impact Point No. 1	31	10	-
Impact Point No. 2	38	49	-
Impact Point No. 3	53	31	-
Impact Point No. 4	78	65.5	-
Impact Point No. 5	78	21	-

**Table 2.** Conditions applied to the performed tests.

**Test No.**	**Mass (kg)**	**Height (m)**	**Energy (J)**
**1**	2.5	0.01	0.245
**2**	2.5	0.02	0.49
**3**	2.5	0.03	0.735
**4**	2.5	0.04	0.98
**5**	2.5	0.05	1.225
**6**	2.5	0.1	2.45
**7**	2.5	0.15	3.675
**8**	2.5	0.2	4.9
**9**	2.5	0.3	7.35
**10**	2.5	0.5	12.25
**11**	2.5	0.75	18.375
**12**	2.5	1	24.5
**13**	10	0.3	29.4

**Table 3.** Residual strain for the mass of 2.5 kg when it is on the Impact Point 1 after the impact.

	**Strain (Microstrains)**	**Distance to the Impact Point (cm)**	**Angle between the FBG and the Line to the Impact**
FBG Sensor 1	5.72	18.86	32°
FBG Sensor 2	−31.08	25.24	11.31°
FBG Sensor 3	−205.92	44.77	32.91°
FBG Sensor 4	15.74	63.91	69.86°
FBG Sensor 5	−13.13	57.76	16.08°
FBG Sensor 6	14.77	54.92	10.49°
FBG Sensor 7	−15.89	24.17	65.55°
FBG Sensor 8	−55.76	35.81	80.91°
FBG Sensor 9	−11.44	77.44	44.74°

**Table 4.** Residual strain for the mass of 10 kg when it is on the Impact Point 1 after the impact.

	**Strain (Microstrains)**	**Distance to the Impact Point (cm)**	**Angle (Related to XY Axis)**
FBG Sensor 1	−10.61	18.86	32°
FBG Sensor 2	−29.37	25.24	11.31°
FBG Sensor 3	−205.57	44.77	32.91°
FBG Sensor 4	30.29	63.91	69.86°
FBG Sensor 5	−10.97	57.76	16.08°
FBG Sensor 6	16.8	54.92	10.49°
FBG Sensor 7	−5.78	24.17	65.55°
FBG Sensor 8	−46.27	35.81	80.91°
FBG Sensor 9	−10.61	77.44	44.74°
